# Peripheral Microangiopathy in Patients with Precapillary Pulmonary Hypertension: Correlation with Cardiac Function and Patients’ Functional Capacity. Study Design and Rationale

**DOI:** 10.31138/mjr.31.3.369

**Published:** 2020-06-10

**Authors:** Alexandra Arvanitaki, George Giannakoulas, Eva Triantafyllidou, Haralambos Karvounis, Theodoros Dimitroulas

**Affiliations:** 1 Fourth Department of Internal Medicine, Hippokration University Hospital, Medical School, Aristotle University of Thessaloniki, Greece; 2 First Department of Cardiology, AHEPA University Hospital, Medical School, Aristotle University of Thessaloniki, Thessaloniki, Greece; 3 Department of Cardiology III - Adult Congenital and Valvular Heart Disease, University Hospital Muenster, Muenster, Germany

**Keywords:** Peripheral microangiopathy, nailfold videocapillaroscopy, precapillary pulmonary hypertension, pulmonary arterial hypertension, systemic sclerosis

## Abstract

Pulmonary hypertension (PH) is a rare, heterogenous clinical entity characterised by a progressive remodelling of pulmonary arterioles, which leads to obstructive pulmonary arteriopathy, increased pulmonary vascular resistance, and eventually, right heart failure. Inflammation, endothelial dysfunction, and microvascular changes of the pulmonary vasculature constitute the hallmarks of pulmonary arterial hypertension (PAH), explaining much of the pathophysiology and clinical manifestations of the disease. Besides pulmonary vasculature, a systemic component of endothelial dysfunction and microcirculation may be involved in PAH, affecting different vascular beds. Nailfold videocapillaroscopy (NVC) is an established method for the assessment of the microvasculature with clinical implications in the diagnostic assessment of individuals with Raynaud syndrome and systemic sclerosis (SSc). Nowadays, growing amounts of evidence suggest that NVC changes in SSc are correlated with other vascular complications such as PAH, supporting a potential link between peripheral and internal organ vasculopathy. The purpose of the current prospective observational study is to explore: 1. the presence of peripheral microangiopathy in precapillary PH using NVC, 2. possible NVC differences among PH subgroups, 3. a potential relationship between NVC morphological abnormalities and clinical, functional, biochemical, echocardiographic and hemodynamic markers of cardiac dysfunction in precapillary PH.

## INTRODUCTION

Precapillary pulmonary hypertension (PH) represents a rare and heterogeneous group of pulmonary vasculopathies defined by elevated mean pulmonary arterial pressure ≥25mmHg, normal pulmonary artery wedge pressure (PAWP) ≤ 15mmHg and elevated pulmonary vascular resistance (PVR) ≥3 Wood Units at rest, that leads progressively to right heart failure and premature death, if left untreated.^1^ Pulmonary endothelial dysfunction and inflammation triggered by shear stress and hypoxia play a vital role in the pathophysiology of pulmonary arterial hypertension (PAH).^2^ All subtypes of PAH such as idiopathic (IPAH), familial PAH, porto-pulmonary PAH, HIV-related PAH, PAH associated with connective tissue diseases (PAH-CTDs), and PAH associated with congenital heart disease (PAH-CHD) share common pathophysiologic features, all of which lead to the progressive remodelling of small and medium sized pulmonary arterioles, and thus to obstructive pulmonary arteriopathy.^2–4^

Research in PAH has been traditionally focused on pulmonary microvasculature, but recently interest has started to divert towards the possibility of peripheral micro-angiopathy. Limited amount of evidence suggests the presence of more generalized endothelial dysfunction in PAH, as determined by forearm blood flow dilation after brachial artery occlusion.^5–7^

Nailfold video-capillaroscopy (NVC) is an established, validated, non-invasive imaging technique for the assessment of the microcirculation, aiding in distinguishing different types of structural and functional microvascular abnormalities.^8^ The most common capillaroscopic parameters are capillary density, capillary dimension, as well as the presence of microhaemorrhages and capillary abnormalities.^9,10^ Thus, NVC has a relevant role in the assessment of Raynaud’s phenomenon and the diagnosis of systemic autoimmune diseases and especially systemic sclerosis (SSc), due to its ability to detect microvascular abnormalities at very early stages.^11–13^ Nailfold video-capillaroscopy has also been investigated in SSc patients with various degrees of vasculopathy, to the point that is considered as a surrogate marker of disease severity and prognosis.^14,15^ In the context of other conditions associated with impaired endothelial function, NVC has been applied in arterial hypertension,^16^ chronic kidney disease,^17^ and diabetes mellitus^18^ to identify the extent of peripheral microangiopathy in these patients. PAH associated with CTDs accounts for 30% of PAH cases.^19,20^ SSc is characterized by skin and internal organ fibrosis, chronic inflammation, autoimmunity activation and microvascular endothelial dysfunction. SSc-associated PAH (SSc-PAH) is one of the most devastating complications of SSc, affecting about 10–15% of patients, and accounts for the high rates of mortality amongst SSc patients with cardiopulmonary involvement.^21^ The presence and severity of peripheral microangiopathy in SSc, as detected using NVC, is considered as an early prognostic marker that determines the risk of developing PH. The correlation between NVC-detected abnormalities and internal organ involvement – predominantly PAH -in SSc subjects, supports the hypothesis that peripheral microvascular changes may be related with structural alterations presenting in pulmonary circulation.^22,23^

Recent studies have compared NVC changes across different subgroups, namely, SSc-PAH individuals, SSc patients without PAH, and healthy controls, and demonstrated a significant reduction in capillary density and a remarkable increase in capillary loop width in SSc-PAH patients,^24,25^ providing an added value to previous reports regarding the presence of a more severe peripheral microangiopathy in SSc-PAH. Furthermore, Hofstee et al. found that the reduction of capillary density was significantly associated not only with the presence, but also with the severity of PAH in both SSc and IPAH individuals,^25^ as it was correlated with hemodynamic parameters. More importantly, a significant decrease in capillary density in IPAH patients was found as compared to healthy controls. These findings were also confirmed by Corrado et al., in 21 IPAH subjects and 20 healthy controls, reinforcing the hypothesis that systemic microcirculatory abnormalities parallel pulmonary microangiopathy in IPAH patients.^24^

Chronic thromboembolic pulmonary hypertension (CTEPH) is also a subtype of precapillary PH that occurs as a result of thrombi non-resolution in pulmonary vascular bed, after at least 3 months of anticoagulation treatment, post-pulmonary embolism.^1^ To date, there are no studies on the presence of peripheral microangiopathy in patients with CTEPH; however, the common pathophysiological mechanisms that share with PAH are a positive indication for the investigation of the presence of systemic vasculopathy in this PH subgroup, as well. Finally, there is also lack of evidence regarding the presence of systemic vasculopathy in PAH-CHD patients.

As a consequence, given the pathophysiological, clinical and functional similarities among the various subtypes of precapillary PH, the evaluation of different capillaroscopic characteristics across the whole spectrum of PH, may provide evidence of a generalized microvascular impairment and indicate a potential link between peripheral and pulmonary microangiopathy in PH.

## METHODS

### Study Design and Setting

This is a prospective case-control observational study, that will be conducted by the Fourth Internal Medicine Department at Hippokrateion General Hospital of Thessaloniki in collaboration with the First Cardiology Department at AHEPA University Hospital. The study received approval from the Aristotle University Ethics Committee, from the Hippokrateion General Hospital Ethics Committee and from the AHEPA University Hospital Ethics Committee. All participants will give informed consent before study entry.

### Inclusion Criteria

This study enrols adult patients with pulmonary hypertension class 1 (Pulmonary Arterial Hypertension) and class 4 (Chronic Thromboembolic Pulmonary Hypertension) according to the classification of 2015 ESC/ERS guidelines.^1^ Patients are either newly diagnosed or currently followed at the Pulmonary Hypertension Unit, 1^st^ Cardiology Department, AHEPA University Hospital and meet the inclusion criteria, based on the hemodynamic definition of 2015 ESC/ERS guidelines (mean pulmonary artery pressure ≥ 25mmHg, pulmonary capillary wedge pressure ≤15mmHg and pulmonary vascular resistance ≥3Woods at rest).^1^ In patients with Eisenmenger syndrome, the diagnosis is based on clinical (cyanosis) and transthoracic echocardiographic (TTE) findings (two-way flow through the defect or right to left shunt) and will not be subjected to right heart catheterization (RHC) as part of the study protocol. Two control groups are enrolled: 1. the healthy control group consists of age- and sex-matched healthy volunteers with the PH patient group; and 2. the SSc control group consists of patients with SSc without PH, monitored in SSc Unit at Hippokrateion General Hospital. PH is excluded in SSc controls by TTE or RHC, if TTE findings are suspicious for the presence of PH.

### Exclusion Criteria

- Patients with precapillary PH that do not belong in Class 1 or 4
- Patients with severe coexisting motion problems, who are unable to complete the 6MWT
- Patients with diabetes mellitus and/or uncontrolled arterial hypertension
- Patients who refuse to complete the informed consent

### Study Overview

All enrolled patients will undergo clinical and paraclinical (invasive and non-invasive) evaluation, in order to confirm PH diagnosis and assess the disease severity, functional capacity and the presence of peripheral microangiopathy. Diagnostic work-up includes: 1. individual and family history; 2. current medication; 3. New York Heart Association (NYHA) functional class; 4. clinical examination; 5. 12-lead electrocardiogram, 6. transthoracic echocardiography (TTE); 7. six-minute walking test (6-MWT); 8. Spirometry; 9. blood tests; 10. NVC; 11. RHC performed in all patients except those with Eisenmenger syndrome. These tests will be performed at the same 1-week time interval for each patient. Healthy controls will undergo medical history, clinical examination and NVC, while SSc controls will undergo additional spirometry and blood tests. Newly diagnosed PH treatment-naïve patients will be re-evaluated with NVC, RHC, 6-MWT and blood tests, 3–9 months after the initiation of PH specific conventional or interventional therapy.

### Study procedures

#### Right Heart Catheterization

The RHC will be performed at the Hemodynamic Laboratory of the 1^st^ Cardiology Department at AHEPA University Hospital of Thessaloniki by specialized medical and nursing staff. Mean pulmonary artery pressure, pulmonary artery wedge pressure, cardiac output, pulmonary vascular resistance, and oxygen saturation in the pulmonary artery will be assessed. Cardiac output is calculated with the method of thermodilution.

#### Transthoracic Echocardiography

Transthoracic Echocardiography will be performed using Vivid S70, General Electric, Norway which is available at the 1^st^ Cardiology Department, AHEPA University Hospital. M-mode and two-dimensional ultrasound study will be performed (determination of the dimensions and functionality of right and left cardiac chambers) as well as classic 2-D Doppler techniques (tricuspid, mitral and aorta flow estimation, maximal velocity and pressure gradient of the tricuspid insufficiency, tricuspid annular plane systolic excursion) based on previous guidelines. The ultrasound parameters will be measured using the Echo Pac GE Healthcare software.

#### 6-MWT

6-MWT will be performed indoors on a 30-meter corridor marked every 1 meter under medical surveillance. Heart rate and arterial saturation will be measured before and after the end of the test. Dyspnoea and fatigue will be assessed with Borg scale before and after the test.^26^

#### Nailfold Video-Capillaroscopy

NVC is an established non-invasive method to assess the capillary vasculature of the digital arteries, which provides information on the structural and functional abnormalities of the capillaries. It is performed at room temperature (22–23°C) with the patient seated and resting for 15 minutes. NVC will be performed at the Fourth Internal Medicine Department at Hippokrateion General Hospital using the Optilia Digital Capillaroscope. At least two adjacent fields of 1 millimeter in the middle of the nailfold finger will be captured from all hands excluding thumbs using the 200x magnification video camera. The images will be analysed with Optipix capillaroscopy software 1.7.x manually by a blinded trained examiner.

NVC parameters to be measured are: capillary density, defined as the number of capillaries in the first row in 1mm (capillaries/mm), capillary width, defined as the maximal diameter of capillary loop, capillary arterial width, capillary venous width, capillary loop width, capillary length (μm), giant capillaries (homogeneously enlarged capillaries > 50μm), capillary ectasias (enlarged capillaries > 20μm and < 50μm), micro-bleeding, oedema, thrombi, any shape abnormalities or disorganization of capillary architecture. All measurements will be performed on the first-row capillaries of each nail. The mean of each capillaroscopic feature will be calculated from the sum of consecutive images for each finger. Subsequently, the average values from eight fingers will be added together and divided by the number of studied fingers.^8,27^ In addition to quantitative and qualitative parameters, a semi-quantitative scoring system will be established to assess the severity of peripheral microangiopathy in PH patients and SSc controls.

#### Blood tests

N-terminal pro-brain natriuretic peptide (NT-proBNP) will be measured in PH patients. A venous blood sample (10 ml) will be centrifuged (serum) and serum measurements will be made on the day of the blood sampling by blinded scientific staff. Haemoglobin, renal function, and immunological tests will also be performed in all PH patients and controls with scleroderma.

#### Spirometry

Spirometry will determine the forced vital capacity (FVC), the forced expiratory volume at first second (FEV1), the ratio FEV1/FVC and the diffusing capacity for carbon monoxide (DLCO%).

## STUDY OUTCOMES

### Primary outcomes

- The presence of peripheral microangiopathy in pre-capillary PH. Comparison of qualitative and quantitative NVC parameters among patients with pre-capillary PH, healthy volunteers and patients with SSc without PH.
- The correlation of NVC parameters with clinical, hemodynamic, echocardiographic, biochemical and functional parameters of disease severity in PH patients.

### Secondary Outcomes

- Assessment of microvascular NVC differences among PH subgroups.
- Assessment of the specific PAH treatment effect on peripheral microcirculation in newly diagnosed PH patients, prospectively.

## SAMPLE SIZE

The precision approach was used for observational studies with 95% confidence intervals. If we consider capillary density (loops/mm) as the main variable for the assessment of peripheral microangiopathy,^24,25^ then the sample of patients with pre-capillary PH required for 95% confidence intervals, with a margin of error δ = ± 0.6mm and standard deviation approximately SD = 1.6mm is calculated by the formula: n≥ (1.96^2^ * SD^2^) / δ^2^ and is 27. With a 10% increase we estimate that we will need at least 30 patients with pre-capillary PH. At least 30 matched healthy volunteers and 30 SSc-non-PH patients are estimated to enter the study.

## STATISTICAL METHODS

Continuous variables are compared using the *t*-test for independent samples or the Mann-Whitney *U* test, while the chi-square test or the Fisher exact test are used to assess categorical variables. For multiple comparisons, one-way ANOVA or the Kruskal-Wallis test with post hoc analysis is used as appropriate. Comparisons are made among PH population, healthy controls and SSc controls. Subgroup analysis will be performed among IPAH, PAH-CTD, PAH-CHD and CTEPH patients, while each subgroup could be compared with healthy controls and SSc controls. Linear regression and logistic regression analysis will be performed to find correlations between NVC parameters, functional, echocardiographic and hemodynamic parameters. A P-value <0.05 is considered statistically significant in this study. Data will be analysed using the SPSS version 26.0.

## ANTICIPATED BENEFITS

The confirmation of the research hypothesis that precapillary PH is characterized by peripheral microangiopathy and its possible association with haemodynamic, echocardiographic, functional, and biochemical parameters may contribute to the establishment of a new non-invasive reliable method – which until recently was mainly applied by rheumatologists - for the assessment of PH disease severity and progression. It will also help in understanding the pathophysiology of the disease. Furthermore, possible changes in peripheral microcirculation after the initiation of PAH-specific treatment will pave the way for the future use of NVC, not only to diagnose early, but also to evaluate treatment effects in patients with precapillary PH. The significance of this non-invasive technique is noteworthy not only because of its impact on medical expenses (lower costs compared to already established **invasive** techniques), but also because of the clear improvement in patients’ quality of care (rapid, painless clinical evaluation of the patient that does not require hospitalization). In addition, the present study is innovative and its results will have an international impact supporting a change in mentality in the overall management of PH patients.
